# Supplementary material for: Neurons in the bat auditory cortex encode class and complexity of future vocalizations
Source: Commun Biol. 2026 May 22;9:699. doi: 10.1038/s42003-026-10319-4 (PMC13197462; doi:10.1038/s42003-026-10319-4)
Supplement: Supplementary file 2 — Supplemental Information [file 42003_2026_10319_MOESM2_ESM.pdf]

Supplementary materials

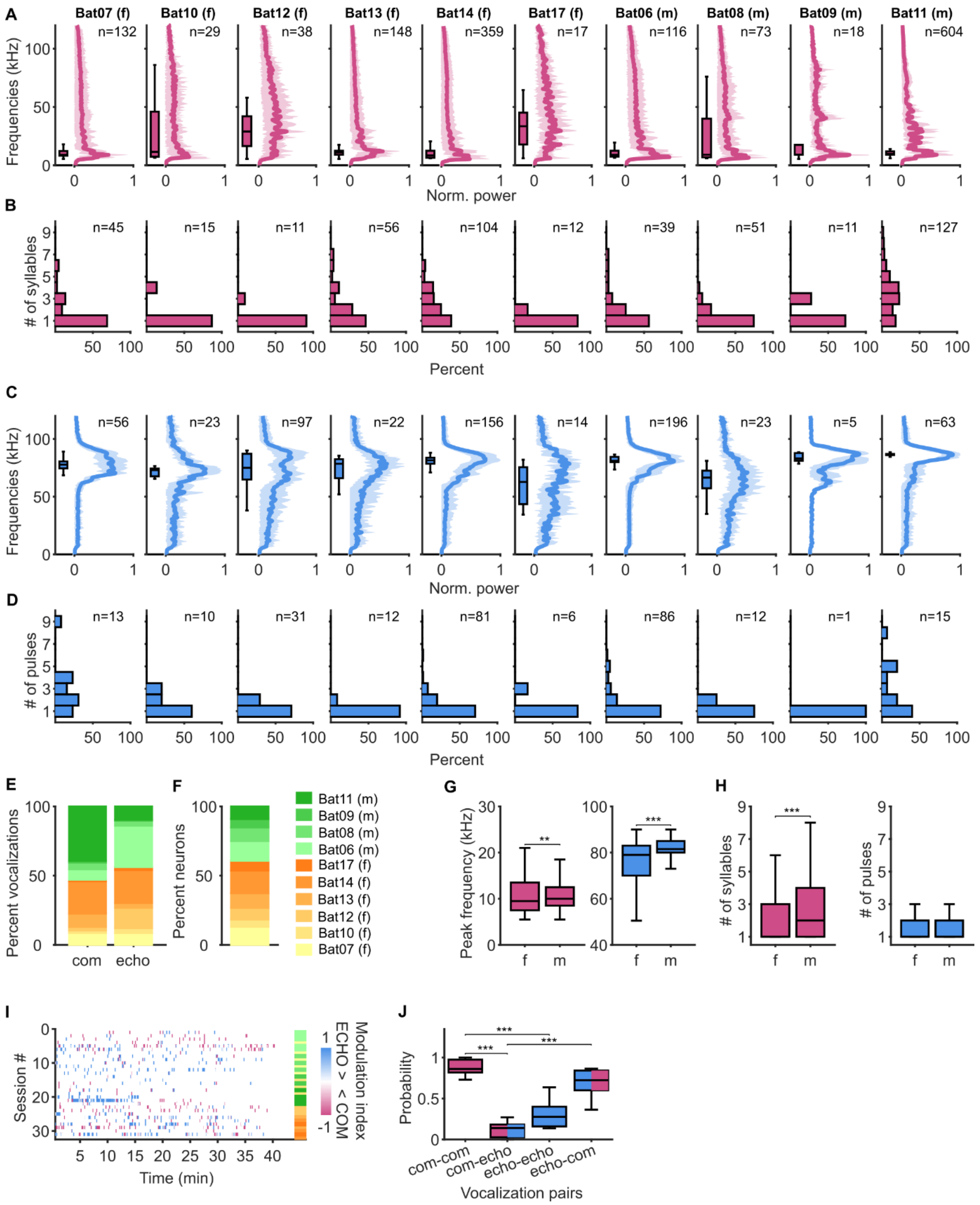

Figure S 1: Vocalizations per individual. A+C, For each individual, peak frequencies (box plots) and power spectra (shaded error plots with mean and standard deviation) for COM (A) and ECHO (C) vocalizations are depicted. The animal ID, the sex and the number of recorded vocalization (i.e. syllables or pulses) is shown on top. B+D, Percentages of vocalizations after a 0.5 s silent period containing a certain number of COM syllables (B) or ECHO pulses (D) for each individual. E, Percent of vocalizations produced by each individual indicated by yellow (female) and green (male) colors. F, Percent of neurons recorded from each individual. G-H, Peak frequencies (G) in COM (left) or ECHO calls (right) and number of COM syllables (left) or ECHO pulses (right) in vocalizations (H) produced

by female vs. male bats. Males have higher peak frequencies and produce more COM syllables. I, Modulation indices indicating ratios of COM vs. ECHO calls in bins of 10 seconds for each recording session over time. Green and yellow colors indicate animal ID of each session as shown in (F). J, Probability of one call type following the other in time bins of 10 seconds. COM syllables are most likely to follow other COM syllables. Results are similar for smaller and larger bin sizes (1 s to 2 min). Box plots show median (line), 25th and 75th percentile (box) and whiskers to the minimum and maximum values within 1.5 times the interquartile range. **$p<0.01$, ***$p<0.001$ Wilcoxon rank-sum test.

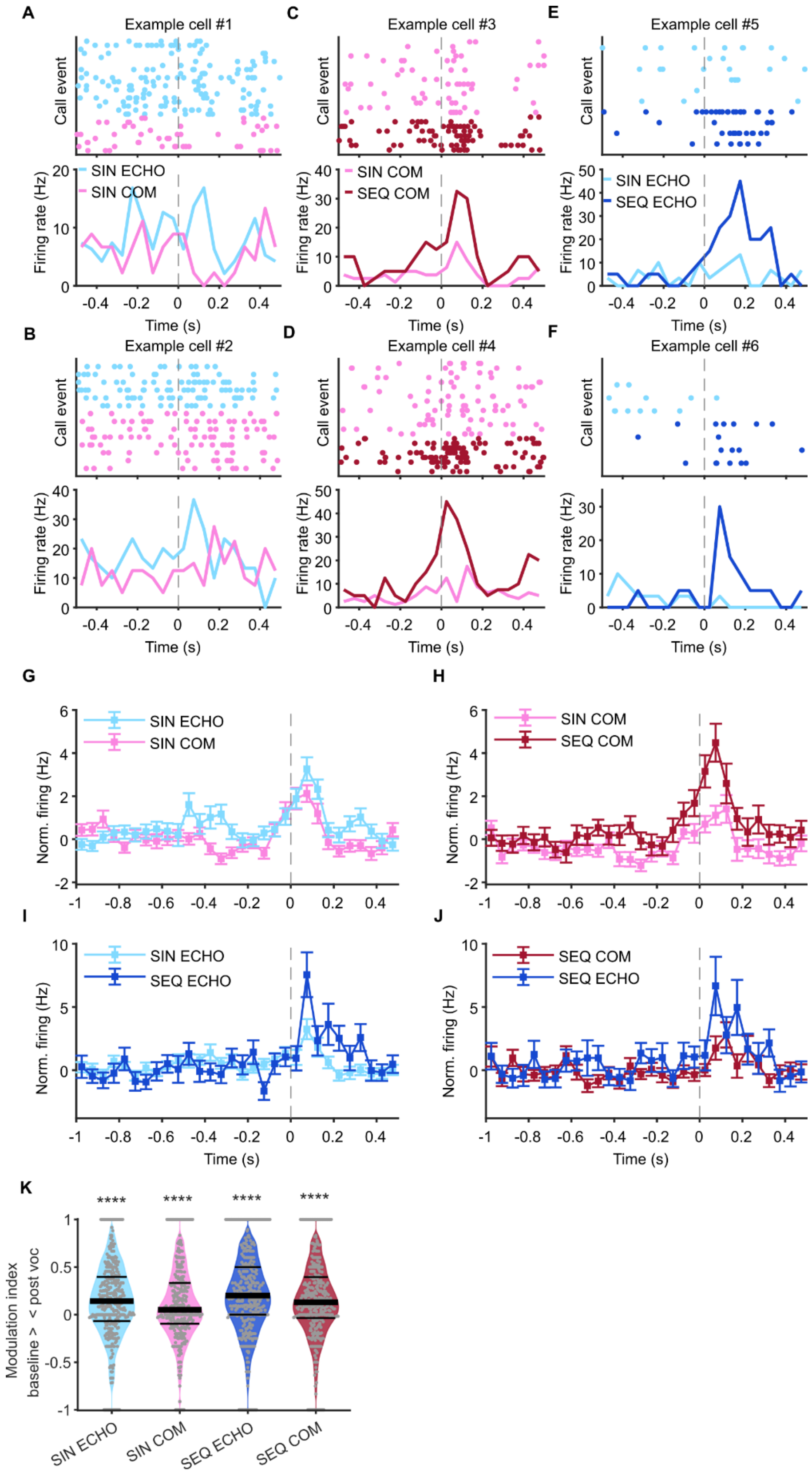

Figure S 2: Example neurons and extended pre-time for neuronal firing rates to different call categories, related to Figure 2. A-F, Example cells recorded during SIN ECHO and SIN COM (A-B), during SIN COM and SEQ COM (C-D), or during SIN ECHO and SEQ ECHO (E-F) with raster plot (top) and averaged firing rates (bottom). G-J, Averaged firing rates as in Figure 2 showing activity 1 s prior to vocal onset. K, Modulation indices for every neuron using activity during baseline (0.5-1 s before vocal onset) and post-vocal window (0-0.5 s after vocal onset) shows significantly increased firing following call onset for every call category. ****p<0.0001 Wilcoxon signed-rank test.

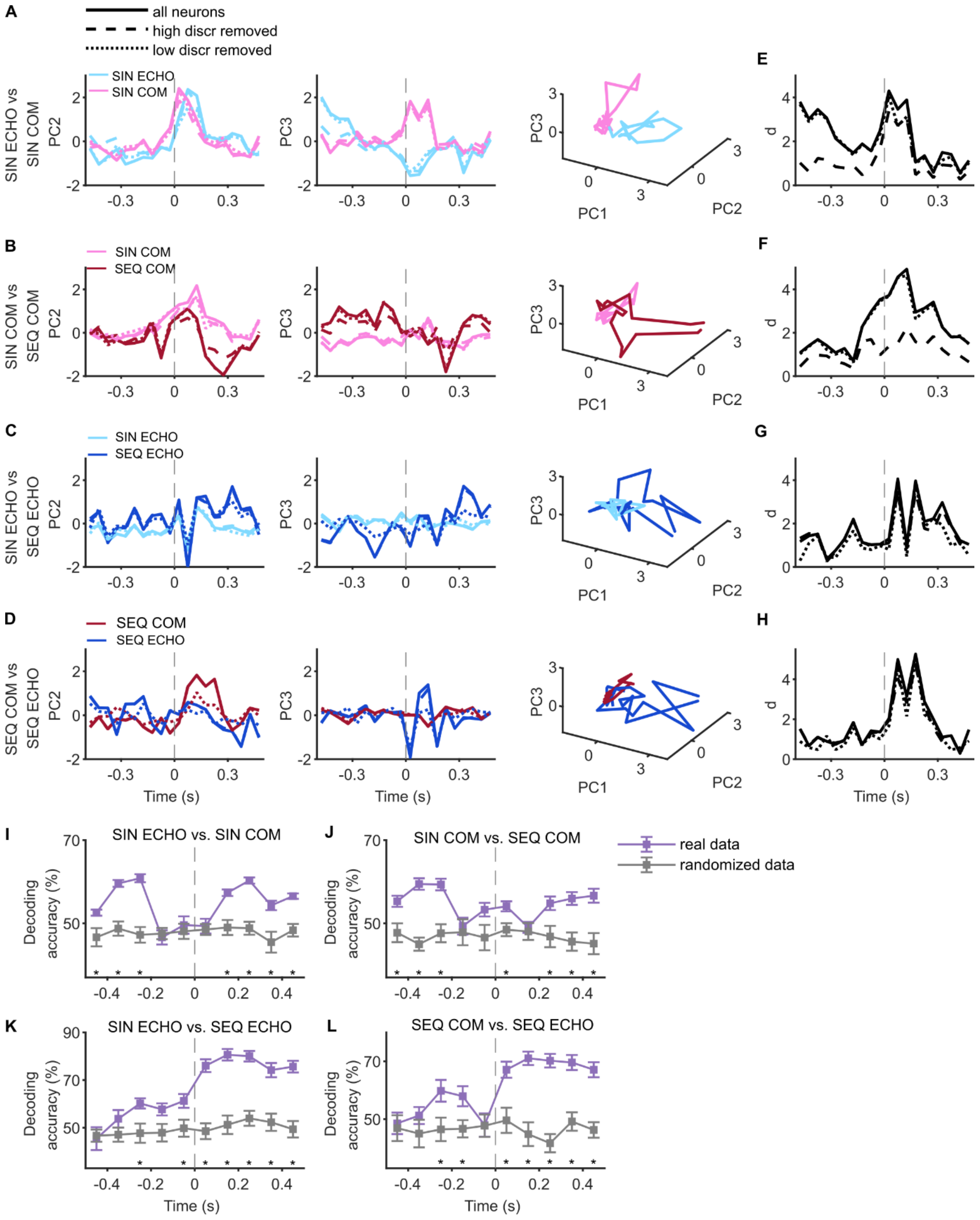

Figure S 3: Additional analysis using neuronal activity to different call categories, related to Figure 2. A-D, 2nd (left) and 3rd (middle) PC scores over time, and 3D plot of PC scores 1-3 (right) from PC analysis on firing rates to call categories of neurons in Figure 2. Solid lines indicate dataset with all neurons, dashed lines indicate dataset without high discriminator neurons (30% of neurons with highest modulation indices), dotted lines indicate dataset without low discriminator neurons (30% of neurons with lowest modulation indices). E-H, Euclidean distance (*d*) of PC 1-3 scores in A-D over time. Line styles as in A-D. I-L, SVM decoding of call category using neuronal activity vectors to all call events from all recorded neurons. *p<0.01 for accuracy using real firing rates vs. randomized dataset, Wilcoxon signed-rank test.

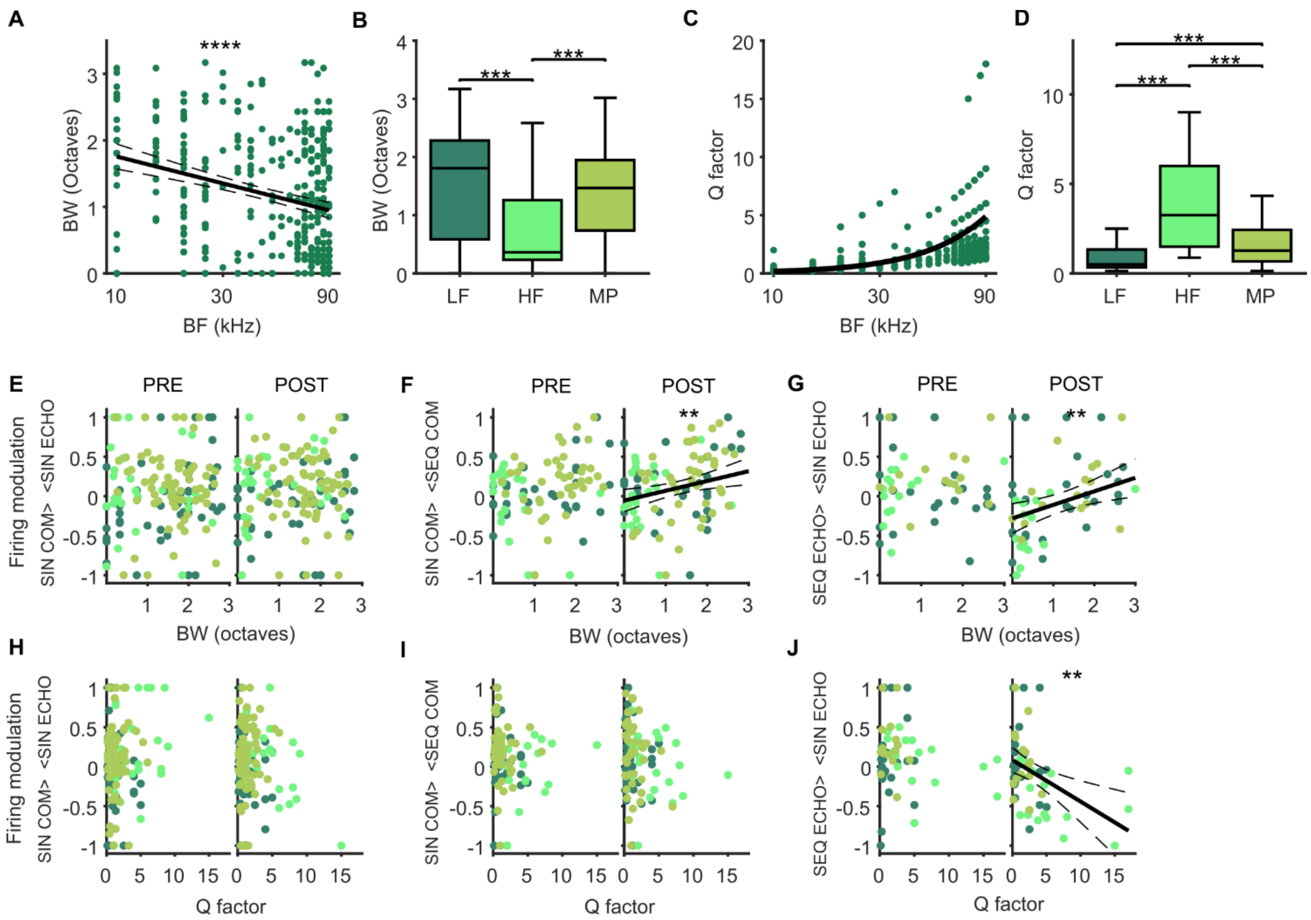

Figure S 4: Additional analysis of frequency tuning properties and relation to call modulation, related to Figure 4 and 5. A, BF and respective BW of every neuron. Solid line indicates linear regression fit, dashed lines indicate 95% CI, asterisks indicate significance of linear regression model with ****p<0.0001. B, BW in LF, HF and MP neurons. C, BF and Q-factor of every neuron. Solid line indicates exponential curve fit. D, Q-factor in LF, HF and MP neurons. Box plots in (B) and (D) represent median (line), 25th and 75th percentile (box) and whiskers extend to the minimum and maximum values within 1.5 times the interquartile range. ***p<0.001, Wilcoxon rank-sum test. E-J, Vocalization-dependent firing modulation index pre (left) and post (right) vocal onset and the respective BW (E-G) or Q-factor (H-J) of each neuron. Modulation indices for SIN ECHO vs. SIN COM (E, H), for SEQ COM vs. SIN COM (F, I) and for SEQ ECHO vs. SIN ECHO (G, J). Solid line indicates linear regression fit, dashed lines indicate 95% CI, asterisks indicate significance of linear regression model with **p<0.01.
